# Inactivation of RNA Viruses by Gamma Irradiation: A Study on Mitigating Factors

**DOI:** 10.3390/v8070204

**Published:** 2016-07-22

**Authors:** Adam J. Hume, Joshua Ames, Linda J. Rennick, W. Paul Duprex, Andrea Marzi, John Tonkiss, Elke Mühlberger

**Affiliations:** 1 Department of Microbiology, School of Medicine, Boston University, 620 Albany Street, Boston, MA 02118, USA; hume@bu.edu (A.J.H.); rennick@bu.edu (L.J.R.); pduprex@bu.edu (W.P.D.); 2 National Emerging Infectious Diseases Laboratories (NEIDL), Boston University, 620 Albany Street, Boston, MA 02118, USA; james218@bu.edu (J.A.); John.Tonkiss@tufts.edu (J.T.); 3 Laboratory of Virology, Division of Intramural Research, National Institute of Allergy and Infectious Diseases, National Institutes of Health, 903 South 4th St, Hamilton, MT 59840, USA; marzia@niaid.nih.gov

**Keywords:** gamma irradiation, virus inactivation, biosafety, BSL-4, vesicular stomatitis virus, measles virus, La Crosse virus, Ebola virus

## Abstract

Effective inactivation of biosafety level 4 (BSL-4) pathogens is vital in order to study these agents safely. Gamma irradiation is a commonly used method for the inactivation of BSL-4 viruses, which among other advantages, facilitates the study of inactivated yet morphologically intact virions. The reported values for susceptibility of viruses to inactivation by gamma irradiation are sometimes inconsistent, likely due to differences in experimental protocols. We analyzed the effects of common sample attributes on the inactivation of a recombinant vesicular stomatitis virus expressing the *Zaire ebolavirus* glycoprotein and green fluorescent protein. Using this surrogate virus, we found that sample volume and protein content of the sample modulated viral inactivation by gamma irradiation but that air volume within the sample container and the addition of external disinfectant surrounding the sample did not. These data identify several factors which alter viral susceptibility to inactivation and highlight the usefulness of lower biosafety level surrogate viruses for such studies. Our results underscore the need to validate inactivation protocols of BSL-4 pathogens using “worst-case scenario” procedures to ensure complete sample inactivation.

## 1. Introduction

Maximum biocontainment laboratories facilitate the study of the most dangerous animal and human pathogens classified as biosafety level 4 (BSL-4) agents. While some experiments may be performed entirely within these facilities, limitations due to space, available equipment, and safety require that some analyses be performed outside of containment using inactivated samples. Sample inactivation is thus of paramount concern, and such procedures are therefore highly regulated at both the local and federal level. In the United States, for example, standard operating procedures (SOPs) for the inactivation of Select Agents (SAs) are subjected to scrutiny by the Centers for Disease Control and Prevention (CDC) Division of Select Agents and Toxins (DSAT) and/or the Animal and Plant Health Inspection Services (APHIS) Agriculture Select Agent Services [1], and require approval by the local Institutional Biosafety Committee (IBC). 

In preparation for Boston University’s National Emerging Infectious Diseases Laboratories (NEIDL) to be permitted to use BSL-4 pathogens, we analyzed the efficacy of specific inactivation techniques that are planned for use to inactivate BSL-4 pathogens. Since a considerable number of BSL-4 pathogens are negative-sense RNA viruses, we chose to use a recombinant (r) vesicular stomatitis virus (VSV) as a surrogate for our initial inactivation testing. Similar to filoviruses and henipaviruses, VSV belongs to the group of nonsegmented negative-sense RNA viruses. However, in contrast to those, it can be manipulated under biosafety level 2 (BSL-2) containment. VSV can be grown to very high titers and causes an overt cytopathic effect (CPE) in infected cells, making it an ideal surrogate virus for evaluating the efficacy of inactivation procedures. Therefore, we generated a recombinant rVSV that encodes the *Zaire ebolavirus* (EBOV) glycoprotein (GP) in place of the VSV GP and contains an additional transcription unit encoding green fluorescent protein (rVSV-EBOVgp-GFP). The use of an rVSV which uses EBOV GP for cell entry better mimics any specific effects that inactivation testing would have on this critical protein. Expression of GFP allows infected cells to be detected by UV microscopy and CPE to be examined by brightfield microscopy. 

To alleviate biosafety concerns, nearly all methods used to inactivate BSL-4 samples are performed entirely within the maximum containment laboratories. The lone exception to this practice is gamma irradiation. For certain analyses, samples containing BSL-4 pathogens need to be inactivated with gamma irradiation in order to preserve the sample for further analysis outside of maximum containment. For a multitude of practical reasons, gamma irradiators are not located within these laboratories and therefore samples containing replication competent pathogens must be transported outside of containment to the irradiators. 

The majority of previous studies using gamma irradiation to inactivate viruses have been limited to dose curves with a given concentration of virus in a particular solute. Inactivation curves of microbes are almost exclusively logarithmic with the exception of a few bacteria that display some initial insensitivity to gamma radiation before the curves become logarithmic [2,3,4,5]. This initial resistance to inactivation by radiation is believed to be mediated by enhanced mechanisms of genomic repair, a mechanism which, to the best of our knowledge, has never been described for viruses [3]. A commonly used method of comparing sensitivity of microbes to gamma irradiation is by comparing the dosage of radiation required to reduce infectivity of a sample by 90% or one log_10_ (D_10_ values) that may be calculated using dose curve plots [6,7]. D_10_ values vary substantially between viruses, although comparison of these values is often complicated by differences in experimental procedures in these studies. Previous work suggested an inverse correlation between viral genome size and susceptibility to inactivation by gamma irradiation [2,8,9,10], although there were exceptions that did not follow this trend [11,12]. 

The mechanism of virus inactivation by gamma irradiation generally falls into two categories: direct and indirect. Direct inactivation of microbes by gamma irradiation is believed to be mainly caused by radiolytic cleavage or crosslinking of genetic material [13,14,15,16,17]. Other targets of direct damage by gamma irradiation include proteins and viral envelopes, although these effects are generally believed to play a minor role in inactivation. Indirect effects of gamma irradiation are principally ascribed to the action of radicals such as ·OH, created from the radiolytic cleavage of water, and to the actions of ozone, created from the radiolytic cleavage of O_2_ to O and its subsequent reaction with another O_2_ molecule. These molecules can react with viral nucleic acids as well as proteins. The destruction of replication competent nucleic acids, via both direct and indirect mechanisms, is believed to be the major mechanism of virus inactivation by gamma irradiation [15,16]. 

The indirect effects of gamma irradiation are dampened by scavengers, molecules that react with hydroxyl radicals and ozone, thereby preventing their ability to act on viral nucleic acids or proteins. While some studies report that proteins in solution (the most common scavengers found in biological samples) have a negative impact on gamma inactivation [6,12,18], other studies suggest that protein content does not alter the efficacy of gamma inactivation, particularly on frozen samples [19,20]. 

In addition to protein content, many other properties of biological samples commonly vary and may have an effect on the ability of gamma irradiation to inactivate samples containing viruses. Decreasing temperature, particularly to the point of sample freezing, has consistently been shown to reduce the effectiveness of gamma inactivation [19,21,22]. However, irradiation of frozen samples potentially offers a higher level of biosafety protection since there is a lower likelihood of leakage from the primary containment vessel. In addition to enhancing biosafety, irradiating samples at lower temperatures has been shown to limit indirect mechanisms of gamma irradiation [6,23,24], potentially reducing the damage to proteins and virion structure while still efficiently inactivating the virus. Therefore, all irradiations performed in this manuscript were carried out on dry ice (−78 °C). 

In order to ensure the effective inactivation of biological samples from maximum biocontainment by gamma irradiation, we aimed to identify factors that mitigate gamma inactivation of rVSV-EBOVgp-GFP. Specifically, we tested variables likely to occur in biological samples commonly produced in BSL-4 laboratories including the effects of viral titers, sample volume, volume of air within sample containers, and total protein content. In addition to ensuring that the samples are effectively inactivated, the biosafety of the samples during transport and irradiation is of paramount importance. Therefore, in combination with the sample tubes being placed within two sealed bags within the leak-proof and shatter-proof transport container, we tested whether the presence of a disinfectant within the sealed bags would have an effect on gamma irradiation. The purpose of the disinfectant would be to immediately inactivate the virus if it leaked from the tube. While rVSV-EBOVgp-GFP was used as a surrogate for filoviruses, we also performed gamma irradiation dose curves and calculated D_10_ values for viruses that serve as surrogates for two other families of BSL-4 pathogens: La Crosse virus (LACV) as a surrogate for bunyaviruses, and measles virus (MV) as a surrogate for henipaviruses. We did this in order to effectively compare the D_10_ values of surrogates for groups of BSL-4 pathogens in a controlled manner. 

## 2. Materials and Methods

### 2.1. Virus Stocks

To generate rVSV-EBOVgp-GFP cDNA, the enhanced GFP (EGFP) gene flanked by the VSV gene start and stop signals was inserted into plasmid pATX-VSVΔG-EBOVgp [25] between the EBOV GP and the VSV L genes using *Xho*I and *Nhe*I restriction sites. For virus recovery, a co-culture of Vero and 293T cells was co-transfected with pATX-VSVΔG-EBOVgp-GFP as well as the four helper plasmids encoding the T7 RNA polymerase and VSV-L, -N, and -P, using Lipofectamine 2000 (Life Technologies, Carlsbad, CA, USA). Then, 72 h after transfection, the supernatant was transferred onto new African green monkey kidney Vero cells (ATCC^®^ CCL81^™^, American Type Culture Collection, Manassas, VA, USA). CPE indicative of rVSV replication was visible at 24–72 h after transfection. rVSV-EBOVgp-GFP stocks were generated as described previously [25]. Briefly, Vero cells were infected at a multiplicity of infection (MOI) of 0.01 and virion-containing supernatants were harvested 2 days post-infection (dpi). Supernatants were clarified by centrifugation at 4500× *g* for 10 min, aliquoted, and stored at −80 °C. Frozen virus was thawed and titrated as described below.

LACV strain H78 (generously provided by J. H. Connor, Boston University, Boston, MA, USA) was grown on baby hamster kidney (BHK) cells (BHK-21 [C-13], ATCC^®^ CCL-10^™^, American Type Culture Collection, Manassas, VA, USA). Briefly, cells were infected at an MOI of 0.01 and supernatants were harvested when cells showed a pronounced CPE. Supernatants were placed on ice for 10 min and then clarified by low-speed centrifugation. Bovine serum albumin (BSA) was added to a final concentration of 10 mg/mL. The virus was titrated as described below and aliquoted and stored at −80 °C. 

Recombinant MV, Khartoum-Sudan strain, containing the EGFP gene as an additional transcription unit (rMV^KS^EGFP(3)) [26,27] was grown in B lymphoblastoid cells (gift from Rik de Swart, Erasmus University Medical Center, Rotterdam, The Netherlands). Briefly, cells were infected at an MOI of 0.01 and the virus was harvested 4 dpi, when CPE and EGFP fluorescence were maximal. Cells and supernatant were subjected to one freeze-thaw cycle to release the cell-associated virus into the supernatant, which was clarified by low-speed centrifugation. The virus was aliquoted and stored at –80 °C. The frozen virus was thawed by incubation at 37 °C and titrated as described below. 

### 2.2. Virus Titration

Viral titers were determined by 50% tissue culture infectious dose (TCID_50_) assay as previously described [28] using the following cell lines: Vero cells for rVSV-EBOVgp-GFP [25], BHK cells for LACV, and Vero cells expressing human CD150 (Vero-hCD150) [29] for rMV^KS^EGFP(3). TCID_50_ quantification was performed using the Spearman–Kärber method [30]. Samples were thawed on ice prior to titration. Virus infection was determined by CPE for LACV and by both CPE and fluorescence for rVSV-EBOVgp-GFP and rMV^KS^EGFP(3). 

### 2.3. Statistics and D_10_ Value Quantification

Statistics were performed using a Student’s *t*-test. D_10_ values were quantified by computing the inverse slope of the regression line best fitting the dose curve of each virus (megarad (Mrad) versus log_10_ TCID_50_ units; regression line calculated in Excel). 

### 2.4. Sample Preparation

Frozen stocks of the indicated viruses were thawed on ice, diluted to a final concentration of 10^6^ TCID_50_ units in a final volume of 1 mL with Dulbecco’s Modified Eagle Medium (DMEM), added to 2 mL tubes (Sarstedt, Nümbrecht, Germany), heat-sealed inside two plastic bags, and then refrozen at –20 °C until used for inactivation unless otherwise stated. All samples were prepared in triplicate. Samples for the analysis of the effect of air volume on inactivation by gamma irradiation were diluted to a final concentration of 10^6^ TCID_50_ units of VSV-EBOVgp-GFP in a final volume of 0.5 mL with DMEM and placed either in 0.5 mL or 2 mL tubes (Sarstedt). Samples for the analysis of the effect of sample media on inactivation by gamma irradiation were diluted to a final concentration of 10^6^ TCID_50_ units of VSV-EBOVgp-GFP in a final volume of 1 mL with DMEM, phosphate-buffered saline (PBS), DMEM plus 10% fetal bovine serum (FBS), or nonhuman primate (NHP) serum and placed in 2 mL tubes (Sarstedt).

### 2.5. Radiation Procedure

All irradiations were performed on dry ice inside of a JL Shepherd Model 484R irradiator (JL Shepherd and Associates, San Fernando, CA, USA) using a cobalt-60 source. For samples requiring long exposures, dry ice was replenished every hour to prevent loss of viability due to temperature increase. Irradiated samples were exposed to 0.5 Mrad of gamma irradiation unless otherwise stated. Non-irradiated samples were left on dry ice as controls. Samples were returned to −20 °C following irradiation. 

## 3. Results

### 3.1. Dose Curves for the Inactivation of Surrogate Viruses

**Figure 1:**
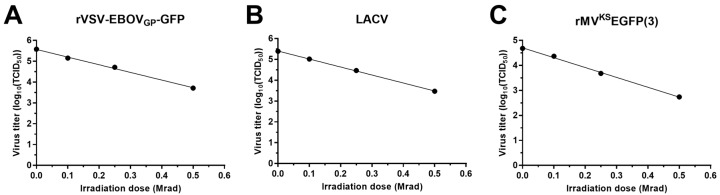
Gamma irradiation of recombinant vesicular stomatitis virus (rVSV) expressing the *Zaire ebolavirus* (EBOV) glycoprotein (GP) and green fluorescent protein (rVSV-EBOVgp-GFP), La Crosse virus (LACV), and recombinant measles virus (rMV), Khartoum-Sudan strain, expressing Enhanced GFP (rMV^KS^EGFP(3)). Samples containing 10^6^ median tissue culture infectious dose (TCID_50_) units of rVSV-EBOVgp-GFP (**A**); LACV (**B**); or rMV^KS^EGFP(3) (**C**) in a volume of 1 mL were subjected to 0.1, 0.25, 0.5, or 3 Mrad of gamma irradiation or left untreated on dry ice. Viral titers were determined by TCID_50_ assay. All samples were completely inactivated with 3 Mrad of gamma irradiation and were therefore not included in the plots used to determine D_10_ values.

Samples of 10^6^ TCID_50_ units of each of the test viruses diluted in 1 mL of DMEM were irradiated with doses of 0.1, 0.25, 0.5 and 3 Mrad or left untreated (Figure 1). All samples irradiated with 3 Mrad were completely inactivated and therefore were not used for the dose response calculations. Using the inverse of the slopes of the regression lines (1/slope) of the dose response to gamma irradiation, the D_10_ value calculated for rVSV-EBOVgp-GFP was 0.271 Mrad, the D_10_ value for LACV was 0.261 Mrad, and the one for rMV^KS^EGFP(3) was 0.253 Mrad. Because comparable D_10_ values were obtained for all these viruses, we focused on rVSV-EBOVgp-GFP for subsequent testing. rVSV-EBOVgp-GFP grows to high titers and causes an obvious CPE in cell culture, which makes it an ideal and easy-to-handle surrogate virus. For all subsequent experiments, we used a dose of 0.5 Mrad as this dose did not completely inactivate rVSV-EBOVgp-GFP, allowing determination of variables that affected the inactivation by gamma irradiation. 

### 3.2. Effect of Virus Concentration on Inactivation by Gamma Irradiation

**Figure 2:**
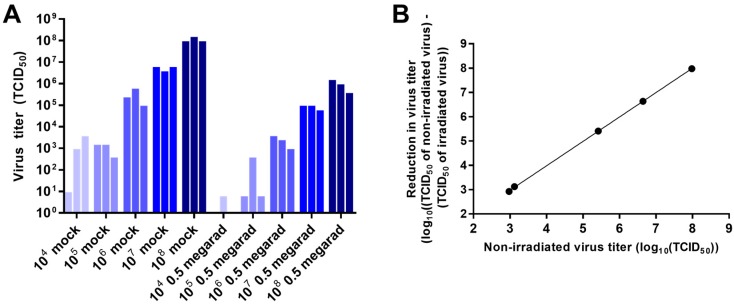
Effect of virus concentration on inactivation by gamma irradiation. (**A**) Samples containing 10^4^, 10^5^, 10^6^, 10^7^, and 10^8^ TCID_50_ units of rVSV-EBOVgp-GFP diluted with Dulbecco’s Modified Eagle Medium (DMEM) to a volume of 1 mL were subjected to 0.5 Mrad of gamma irradiation or left untreated (mock) on dry ice. Viral titers were determined by TCID_50_ assay; (**B**) Plot of non-irradiated sample titers versus the reduction of titer following 0.5 Mrad (log_10_((non-irradiated sample titer) – (irradiated sample titer)), means of data from (**A**)). Regression line equation shown. Data represent three independent experiments.

In order to test the effect of virus concentration on inactivation by gamma irradiation, samples of rVSV-EBOVgp-GFP containing 10^4^, 10^5^, 10^6^, 10^7^, or 10^8^ TCID_50_ units diluted in 1 mL of DMEM were irradiated with a dose of 0.5 Mrad. Figure 2A presents the inactivation of rVSV-EBOVgp-GFP following gamma irradiation as a function of initial virus concentration. In line with previous dose response data [18], the reduction of rVSV-EBOVgp-GFP was directly proportional to the initial concentration of the virus (Figure 2B). Variability observed in replicate samples is likely due to the effect of sample freezing and thawing, differences in inactivation caused by the stochastic nature of the irradiation, or both of these effects. These differences appear greater in samples containing small amounts of virus due to the log_10_ transformation of the data. 

### 3.3. Effect of Sample Volume on Inactivation by Gamma Irradiation

**Figure 3:**
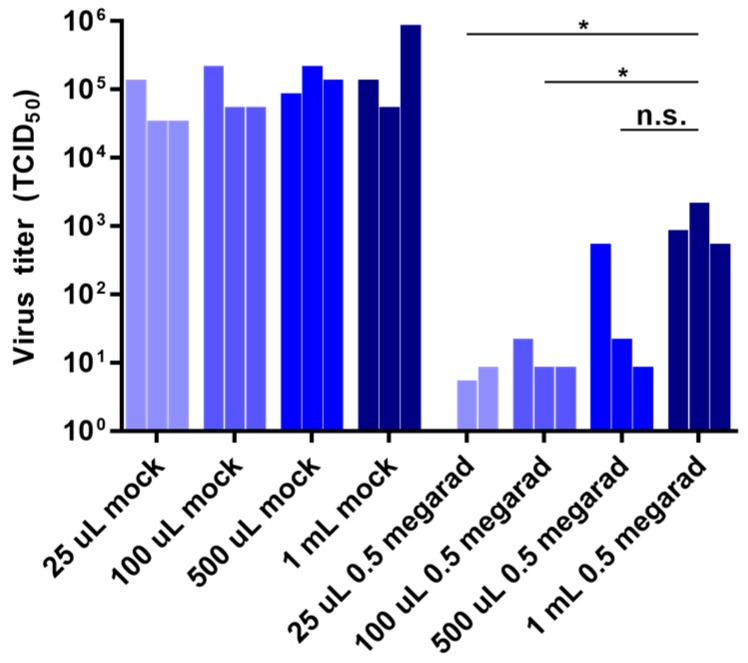
Effect of sample volume on inactivation by gamma irradiation. Samples containing 10^6^ TCID_50_ units of rVSV-EBOVgp-GFP diluted with DMEM to final volumes of 25 µL, 100 µL, 500 µL, and 1 mL were subjected to 0.5 Mrad of gamma irradiation or left untreated (mock) on dry ice. Viral titers after were determined by TCID_50_ assay. Data represent three independent experiments. * *p* < 0.05; n.s., not significant.

Samples originating in BSL-4 laboratories commonly come in a diversity of volumes. Because of this variability and its unknown influence on irradiation, we compared the effect of initial sample volume on the inactivation of rVSV-EBOVgp-GFP by gamma irradiation. Briefly, equal amounts of rVSV-EBOVgp-GFP were diluted with DMEM into sample volumes of 25 µL, 100 µL, 500 µL, and 1 mL and exposed to 0.5 Mrad of gamma irradiation. As shown in Figure 3, rVSV-EBOVgp-GFP was more resistant to inactivation by gamma irradiation in larger sample volumes. These results demonstrate that the volume of samples being irradiated has an effect on inactivation, a factor not previously recognized as playing a role in inactivation by gamma radiation. 

### 3.4. Effect of Air Volume on Inactivation by Gamma Irradiation

**Figure 4:**
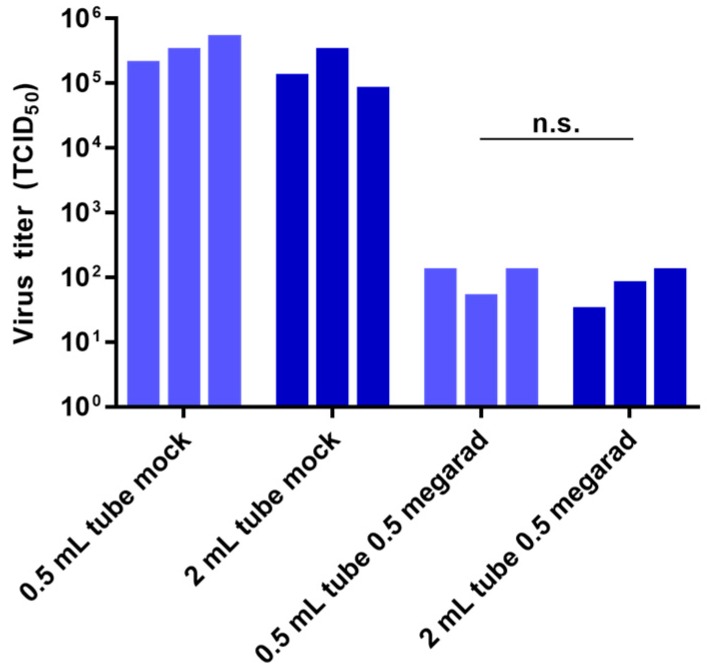
Effect of air volume on inactivation by gamma irradiation. Samples containing 10^6^ TCID_50_ units of rVSV-EBOVgp-GFP diluted with DMEM in a final volume of 500 µL were placed in each 0.5 mL and 2 mL screwcap tubes and were subjected to 0.5 Mrad of gamma irradiation or left untreated (mock) on dry ice. Viral titers were determined by TCID_50_ assay. Data represent three independent experiments. n.s., not significant.

Inactivation by gamma irradiation is partly due to the radiation-induced creation of ozone within the air of samples [23,31]. There is some evidence that the indirect mechanisms of inactivation by gamma irradiation, which includes the effects of ozone, is diminished in frozen samples [23]. Since all of the samples we tested were frozen, it was unclear if the volume of air within the samples would have an effect on gamma irradiation-induced inactivation. Therefore, we examined the effect of gamma irradiation on rVSV-EBOVgp-GFP diluted in a volume of 500 µL DMEM placed either in 0.5 mL or 2 mL screwcap tubes with air volumes of approximately 0.55 cm^3^ and 1.87 cm^3^, respectively. No statistically significant difference (determined by Students *t*-test, *p*-value = 0.296) was observed in the ability of gamma irradiation to inactivate samples in tubes with differing air volumes (Figure 4). These data indicate that the volume of air within sample containers has a negligible effect on the inactivation of frozen samples of rVSV-EBOVgp-GFP by gamma irradiation and suggest that, contrary to studies done at room temperature or 4 °C, ozone does not play a role in this mode of inactivation. 

### 3.5. Effect of Media Composition on Inactivation by Gamma Irradiation

**Figure 5:**
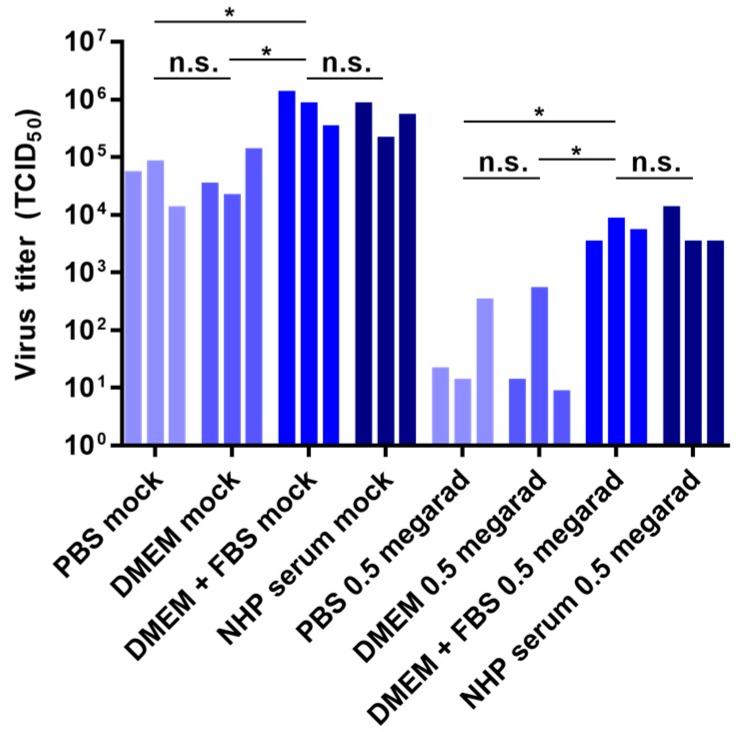
Effect of sample media on inactivation by gamma irradiation. Samples containing 10^6^ TCID_50_ units of rVSV-EBOVgp-GFP diluted to a final volume of 1 mL with DMEM, DMEM containing 10% fetal bovine serum (FBS), phosphate-buffered saline (PBS), or nonhuman primate (NHP) serum were subjected to 0.5 Mrad of gamma irradiation or left untreated (mock) on dry ice. Viral titers were determined by TCID_50_ assay. Data represent three independent experiments. * *p* < 0.05; n.s., not significant.

Previous reports provide conflicting data regarding the effect of media content on viral inactivation by gamma irradiation, particularly with frozen samples. Therefore, we tested the effect of diluent protein content on viral inactivation by gamma irradiation using rVSV-EBOVgp-GFP. Samples of 10^6^ TCID_50_ units of rVSV-EBOVgp-GFP diluted in 1 mL of each PBS, DMEM, DMEM containing 10% FBS, or NHP serum were irradiated with 0.5 Mrad of gamma radiation or left untreated. Diluent protein content had an effect on both the non-irradiated samples as well as the irradiated samples: viral titers were significantly higher in samples diluted in FBS-containing DMEM or NHP serum than samples diluted in PBS or DMEM (Figure 5). The effect of protein content in the non-irradiated samples is likely due to a stabilizing effect on the freeze-thaw of the samples [32], while the effect of protein content on the irradiated samples is likely due to a combination of stabilization during freeze-thaw as well as the free radical scavenging properties of the proteins in the samples. 

### 3.6. Effect of External Disinfectants on Inactivation

**Figure 6:**
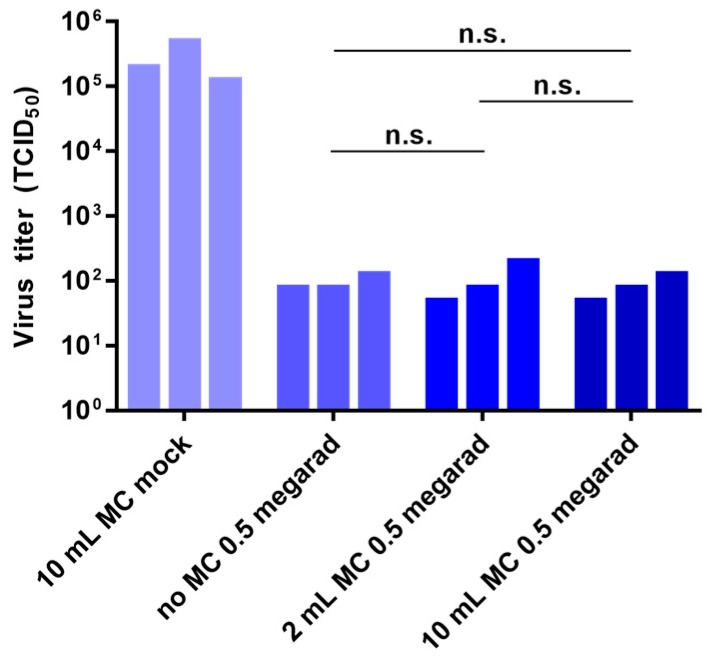
Effect of external disinfectant on inactivation by gamma irradiation. For the study, 2-mL tubes containing 10^6^ TCID_50_ units of rVSV-EBOVgp-GFP diluted to a final volume of 1 mL with DMEM were each placed in two small, concentric, heat-sealed bags containing no MicroChem-Plus (“no MC 0.5 Mrad”), 1 mL of MicroChem-Plus per bag (“2 mL MC 0.5 Mrad”), or 5 mL of MicroChem-Plus per bag (“10 mL MC 0.5 Mrad”) and were subjected to 0.5 Mrad of gamma irradiation. Sample tubes were also placed in two small heat-sealed bags containing 5 mL of MicroChem-Plus per bag (“10 mL MC mock”) and left on dry ice. Viral titers were determined by TCID_50_ assay. Data represent three independent experiments. n.s., not significant.

Since gamma irradiators are not located within BSL-4 laboratories, live samples are sealed in double plastic bags and transported in leak-proof, shatter-proof containers to the irradiators outside of containment. To further enhance the biosafety while transporting these samples, we tested whether the addition of a disinfectant to the sealed pouches surrounding the samples would interfere with inactivation by gamma irradiation. Samples of 10^6^ TCID_50_ units of rVSV-EBOVgp-GFP diluted in 1 mL of DMEM were packaged either in empty double plastic pouches or in pouches containing 1 mL or 5 mL of MicroChem-Plus (National Chemical Laboratories, Inc, Philadelphia, PA, USA) per pouch. MicroChem-Plus is a quaternary ammonium compound commonly used in BSL-4 laboratories to inactivate BSL-4 viruses. The samples were either not irradiated (the non-irradiated samples contained 5 mL MicroChem-Plus per pouch) or irradiated with 0.5 Mrad of gamma radiation. Addition of external MicroChem-Plus to the pouches containing the samples had no effect on the inactivation of rVSV-EBOVgp-GFP (Figure 6). 

## 4. Discussion

Due to the stochastic nature of using radiation to inactivate biological samples, there is no dosage that can be considered to inactivate 100% of a sample. Instead, a probabilistic measure, the sterility assurance level (SAL), is frequently used to describe the likelihood that a single infectious pathogen remains in an irradiated sample [33]. For example, if a sample to be irradiated contained 10^6^ TCID_50_ units and the SAL for the pathogen is set at 10^−6^ (meaning that that there is one chance in a million that the sample retains a single infectious particle), then a 12 log reduction in pathogen titer is needed to achieve the SAL. If the D_10_ for the pathogen is 0.25 Mrad, it would require a dose of 3 Mrad (0.25 Mrad × 12). Doubling the gamma irradiation dose to 6 Mrad would raise the SAL to 10^−18^. Considering the starting virus titers that we used in this study of 10^6^ TCID_50_ units, it is not surprising that the 3 Mrad dosage completely inactivated all of our samples since our calculated D_10_ values would achieve an SAL of 8.51 × 10^−6^ for rVSV-EBOVgp-GFP, 3.20 × 10^−6^ for LACV, and 1.39 × 10^−6^ for rMV^KS^EGFP(3) with such a dose. An SAL commonly used for the inactivation of pathogens of concern is 10^−6^, although this may need to be modulated based on risk assessment [33]. 

Factors that alter the D_10_ value by mitigating the ability of gamma irradiation to inactivate a pathogen have a direct impact on both the SAL achieved with a given dose of irradiation as well as the dose of irradiation required to achieve a specific SAL. We identified two factors, sample volume and solute protein content, which alter the D_10_ value by compromising the ability of gamma irradiation to inactivate frozen rVSV-EBOVgp-GFP-containing samples. Another tested variable, virus concentration, alters the dosage required to achieve a specific SAL but appears not to alter the rate at which viruses are inactivated. Air volume and the presence of external inactivating agent had no impact on inactivation by gamma irradiation. 

Consequently, while performing tests on the ability of gamma irradiation to inactivate viruses, it would be sensible to use “worst case scenario” samples containing the highest viral concentration, sample volume, and protein content likely to be found in samples generated within a given laboratory. Any samples containing a higher virus concentration, a larger volume, or a higher protein content than those used in experimentally-validated tests would therefore require additional testing prior to them being considered safely inactivated. Testing under different conditions (e.g., irradiating at a different temperature) may require the re-testing of certain variables. For instance, the air volume of a sample may lead to enhanced viral inactivation when samples are irradiated at non-freezing temperatures due to the possibility of gamma radiation-created ozone contributing to viral inactivation [31]. 

The addition of external MicroChem-Plus around the samples did not alter inactivation of rVSV-EBOVgp-GFP by gamma irradiation. Having the sample tubes surrounded by MicroChem-Plus during the transport to the irradiator and while being irradiated represents a useful safety precaution since any virus which leaked would immediately come in contact with a disinfectant [34]. Thus, the addition of this chemical to sample containers provides enhanced biosafety while the samples are being arranged for irradiation without altering the dosage required to achieve the desired SAL. 

Reported susceptibilities of viruses to inactivation by gamma irradiation sometimes vary, as seen by looking at their gamma irradiation D_10_ values (see [33] for a thorough listing). Published D_10_ values, which are often inconsistent, are likely dissimilar due to differences in protocols used for the testing (e.g., different sample volumes, solute contents, temperatures, etc.). Nevertheless, closely related viruses typically have similar sensitivities to inactivation by gamma irradiation when performed using similar experimental protocols (see Table 8 of [33]). BSL-4 pathogens are nearly all enveloped, single-stranded RNA viruses and are therefore likely to have similar D_10_ values. In support of this hypothesis, the three single-stranded RNA viruses we tested, including a bunyavirus (LACV), a rhabdovirus (rVSV-EBOVgp-GFP), and a morbillivirus (rMV^KS^EGFP(3)), all had similar D_10_ values when analyzed using the same experimental protocol. Therefore, the results published here, as well as future irradiation studies using appropriate BSL-2 surrogate viruses, can be used to extrapolate what is likely to be the case for viruses that are restricted to study in maximum containment facilities. This work in particular highlights the potential for use of rVSV-EBOVgp-GFP as a surrogate for filoviruses in such irradiation studies, as it shares many characteristics with these viruses—it is a nonsegmented negative-sense RNA virus that grows to high viral titers and encodes EBOV GP—but can be studied under BSL-2 conditions. However, although it is likely that the D_10_ values of negative-sense RNA BSL-4 viruses are similar to those determined in this study, this remains to be confirmed by inactivation studies using select BSL-4 viruses representing different families of BSL-4 pathogens (e.g., EBOV as a representative of filoviruses).
